# Birefringence effect studies of collagen formed by nonenzymatic glycation using dual-retarder Mueller polarimetry

**DOI:** 10.1117/1.JBO.27.8.087001

**Published:** 2022-08-04

**Authors:** Chi-Hsiang Lien, Zong-Hong Chen, Quoc-Hung Phan

**Affiliations:** National United University, Department of Mechanical Engineering, Miaoli, Taiwan

**Keywords:** birefringence, collagen, nonenzymatic glycation, Mueller polarimetry

## Abstract

**Significance:**

Nonenzymatic glycation of collagen covalently attaches an addition of sugar molecules that initially were involved in a reversibly reaction with amino groups on the protein. Due to the ultimate formation of stable irreversible advanced glycation end products, the process of glycation leads to abnormal irreversible cross-linking, which ultimately accumulates with age and/or diabetes in the extracellular matrix, altering its organization.

**Aim:**

We report the use of dual-retarder Mueller polarimetry in conjunction with phase retardance to differentiate collagen cross-linking in a normal collagen gel matrix from that in tissues with nonenzymatic cross-linking.

**Approach:**

A dual-liquid crystal-based Mueller polarimetry system that involves electronic modulation of polarization state generators (PSGs) was employed to produce all types of polarization states without moving any part and enable detection of the signal directly using a Stokes polarimeter. The linear phase retardance response was obtained for the characterization of the solution and gel forms of collagen using differential Mueller matrix analysis.

**Results:**

We found that linear phase retardance measurements via differential Mueller matrix polarimetry successfully differentiated collagen gel matrices with different degrees of cross-linking formed by a nonenzymatic glycation process and demonstrated that this technology constitutes a quick and simple modality.

**Conclusions:**

This approach has high sensitivity for studying differences in fibrillar cross-linking in glycated collagen. Further, our work suggests that this method of structural analysis has potential clinical diagnostic value owing to its noninvasive and cost-efficient nature.

## 1 Introduction

Collagen is the most abundant extracellular matrix (ECM) protein in the human body by weight and is critical for various tissues, for it functions as both a structural component and a signaling molecule operating via biomolecular interactions. Collagen constitutes 30% of ECM proteins and interacts with a diverse range of biomolecules, including proteins, sugars, proteoglycans, polyphenols, and drugs.^1^ A number of studies have investigated the influence of ECM mechanics on several diseases, such as cancer, corneal diseases, osteoarthritis, connective tissue diseases, autoimmune disorders, and cardiovascular diseases,^2^ showing fibrous changes occurring in the microenvironment of tissues at the molecular level. Several studies have demonstrated that collagen protein molecules can covalently add sugar molecules not only through a natural enzyme-catalyzed reaction but also by a nonenzymatic reaction called glycation. Recent studies have indicated that the nonenzymatic glycation of collagen occurs with the accumulation of collagen in the ECM, with age and/or diabetes, leading to alteration of tissue mechanics and cell function. The end products of these reactions are known to be permanent. Therefore, the effects of nonenzymatic cross-linking on collagen are important factors to be studied in modern biomaterial science because they affect the properties of tissues or biologically derived scaffolds and play an essential role in diseases. Effects of nonenzymatic cross-linking are also implicated in the pathology associated with diabetes, atherosclerosis, and Alzheimer’s disease.^3^^–^^6^ Yuen et al. reported the effect of diabetes-induced glycation of the ECM on cell function by modeling the glycation reaction based on collagen treated with methylglyoxal for elucidating the development of fibrosis in diabetes.^7^ Collagen glycation was thus shown to enhance intercellular adhesion formation and cell migration over collagen to generate myofibroblasts.

**Fig. 1:**
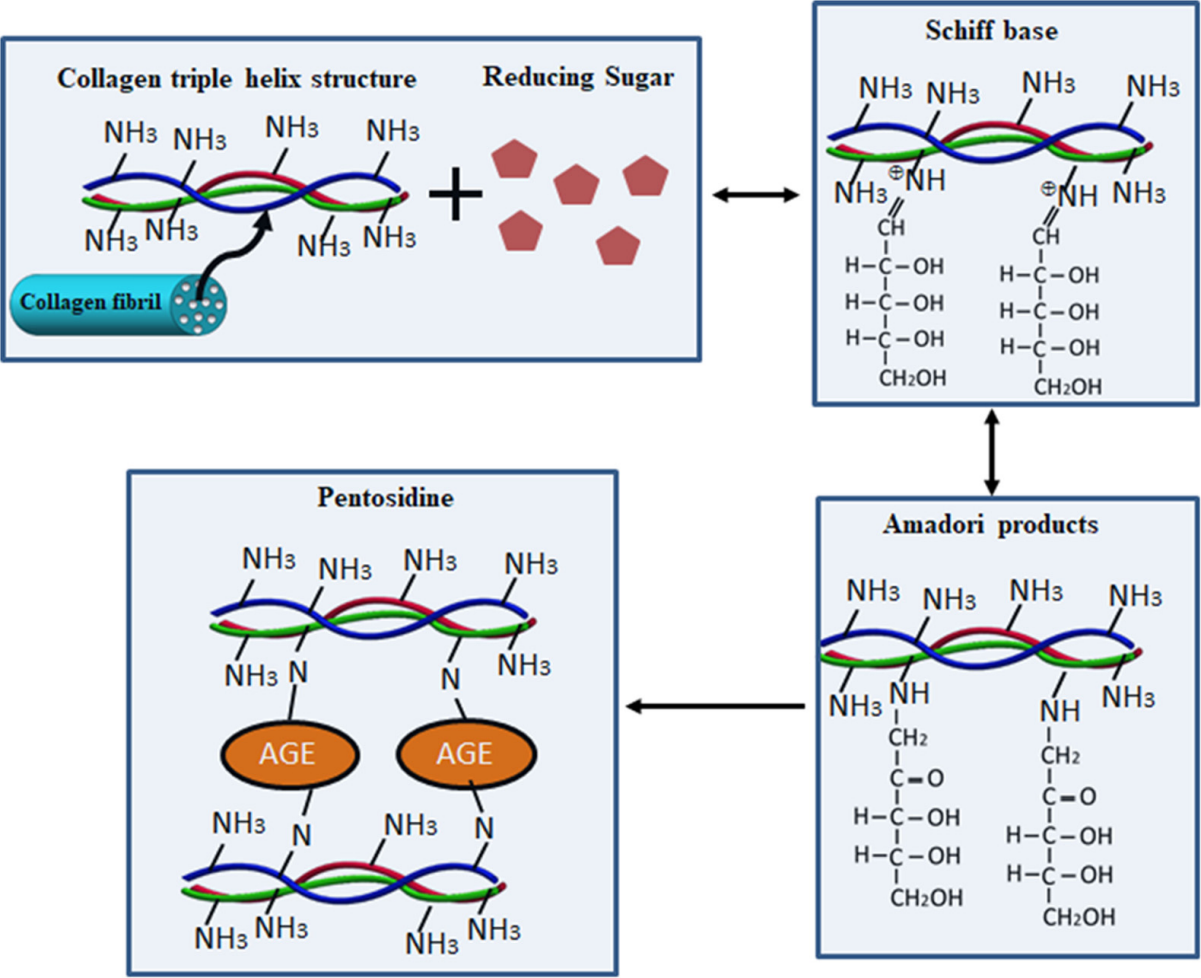
Major mechanism of collagen glycation.

At the molecular level, the major mechanism of glycation involves the reversible formation of Schiff bases that are reversibly transformed into Amadori adducts on collagen proteins, leading to the ultimate irreversible formation of stable advanced glycation end products (AGEs), as shown in Fig. 1. Recent cutting-edge research has established a variety of methods to evaluate collagen protein glycation based on the detection of AGEs or collagen molecular changes.^8^^–^^11^ Some of these methods, such as antibody detection,^12^ fluorescence spectroscopy,^9^^,^^13^^,^^14^ Fourier transform infrared (FTIR) spectroscopy,^10^ and multiphoton^13^^,^^15^ and confocal Raman microscopy,^3^^,^^16^^,^^17^ are only applicable directly to *ex vivo* or *in vivo* samples. Other methods for the quantitative determination of intermolecular protein cross-linking, such as electrophoresis, mass spectrometry, and high-performance liquid chromatography, have largely been limited to nonroutine specialized applications owing to the highly technical sample preparation required for these methods.^18^

The above-mentioned study by Rancis-Sedlak et al. shows Col I gel exhibiting statistically significant increases in autofluorescence, cross-linking, and resistance to proteolytic degradation in proportion to increasing the concentration of a reducing sugar (glucose-6-phopshate) in the gel for an incubation period of 5 days.^8^ They demonstrated that the increase in the reducing sugar concentration was associated with changes in the mechanical properties of collagen gels. This phenomenon led to an increase in the protein volume fraction and the storage and loss moduli of Col I gel, which, in turn, caused an increase in cross-linking in the intermolecular spacing of collagen during glycation. Glycated collagen gels are seen to have similar morphology (fiber density and orientation) when observed using confocal microscopy and intensity signals are seen to decrease in glycated collagen gels compared with those of control gels when observed using SHG microscopy, as demonstrated by Kim et al.^15^ Herein, the network morphology of the Col I gel matrix is sensitive to the varying condition parameters during self-assembly into fibrils, such as the concentration of collagen monomers, temperature, pH, and ionic strength. According to early studies for quantitative molecular analysis, they found a decreased pore size and growing turbidity with increasing collagen gel concentrations (0 to 5 mg/ml), which may result from increasing the number, increasing the randomness distribution of fiber orientation, and/or increasing the size of collagen fibrils.^19^^–^^22^

Collagen fibrils, being complex molecular structures, have intrinsic linear birefringence arising due to anisotropy at the molecular scale, as well as to the anisotropy of form, and the overall linear birefringence is fundamentally determined by the anisotropic distribution of spatially electrical charges. Previous studies have used polarized light to detect and analyze the anisotropic properties of collagen using tissue samples. Boulesteix et al.^23^ used a polarimetric imaging system to measure the Mueller matrices of stained collagen samples. In addition, the investigation of the comparative study of Mueller and SHG imaging for uterine cervical cones and vaginal tissues is reported by Schanne-Klein research group.^24^^,^^25^ The results indicated that Mueller’s approach allowed for the reconstruction of collagen fiber orientation maps to expand their ability to acquire microstructural information. Pham et al.^26^ also demonstrated the algorithm to extract the effective parameters of anisotropic materials based on the decomposition formalism proposed by Lu and Chipman.^27^ The problem of the strict sequential order of matrix components required for the decomposition method was resolved by a differential Mueller matrix proposed by Azzam et al. and Ortega-Quijano and Arce-Diego,^28^^,^^29^ and the determination of the anisotropic parameters was enabled by an approach presented by Liao and Lo.^30^ Researchers have increasingly been attempting to develop the novelty quantification approach to provide a better understanding for biological samples, specifically for collagen-rich tissue. Dong et al.^31^ proposed the backscattering Mueller matrix configuration setup using the efficient calculation Mueller matrix transformation method and demonstrated that it is suitable for monitoring the microstructural changes of skin tissues during UVR-induced photodamaging. In transmission configuration using the logarithmic Mueller matrix decomposition (LMMD) algorithm, Lee et al.^32^ found that total linear retardance of the dermal layer containing collagen and elastic fibers depended linearly on thickness and that the increase of standard deviation of the retardance values varied with tissue thickness because of the thickness fluctuations. Based on Beer–Lambert law, some quantitative ratios of parameters, which are thickness invariant, were proposed to provide the higher contrast mapping biological images to minimize the thickness fluctuation effects. Furthermore, Li et al.^33^ used Mueller matrix microscopy data,^32^ the logarithmic decomposition approach, and polarized Monte Carlo (MC) modeling for analyzing tissue microstructure in human skin equivalents. Recently, Lee et al.^34^ demonstrated that the images of polarization and depolarization parameters with LMMD are available for collagen visualization of cervical tissues using a liquid crystal-based Mueller polarimetric imaging system. The results for the mice model of pregnancy indicated that this method has a good potential to become a clinical tool for *in vivo* detection of the PTB risk. Therefore, Stokes–Mueller matrix polarimetry is a label-free, direct, and nondestructive optical method that can measure molecular organization in biological samples under their native conditions.

A better understanding of the characteristics of biomolecules at the level of microstructures is necessary to clarify the effects of nonenzymatic cross-linking on tissues and biologically derived scaffolds. To address the challenges posed in the understanding of the interactions involved in collagen glycation, herein, we report the production of native, fibrillar, and nonpepsined type I collagen glycated *in vitro* using ribose as a reducing sugar. Moreover, this study is the first to demonstrate the use of the differential Mueller matrix polarimetry approach for the detection and analysis of glycated fibrillar collagen. Our investigation shows that this method is a relatively simple modality that could facilitate structural analyses of biological tissues at the macromolecular level for performing noninvasive collagen measurements for clinical diagnostic applications.

## 2 Materials and Methods

### 2.1 Sample Preparation

The collagen (type I) solution, obtained from a rat tail (354249, Corning), was used to prepare fiber samples grown *in vitro*. For the analysis of the collagen matrix, each series of comparisons of collagen was performed with gels polymerized at the same time. Three experimental replicates were used to determine the appropriate sample size. For all measurements, each gel had three individual data to provide the average and standard deviation of the optical parameter. A collagen solution having a working concentration of 1 to 2 mg/mL was prepared on ice and diluted with a phosphate-buffered saline (PBS) solution to constitute the total gel volume. The pH of the collagen solution was neutralized using sterilized sodium hydroxide (NaOH). The collagen solution was slowly polymerized in an Ibidi μ-slide 8-well (Ibidi GmbH, Germany) culture chamber at 4°C for 20 h. The gels, with thicknesses of 0.5 to 1.5 mm, were released from the bottom of the sides of the dish and then fixed in 4% paraformaldehyde. Glycated collagen was obtained by incubating collagen gels (2 mg/mL) at 37°C for 4 days with different concentrations of D-ribose (R7500, Sigma) in 1× PBS and buffered to physiological pH at the same temperature.

### 2.2 Experimental Setup

**Fig. 2:**
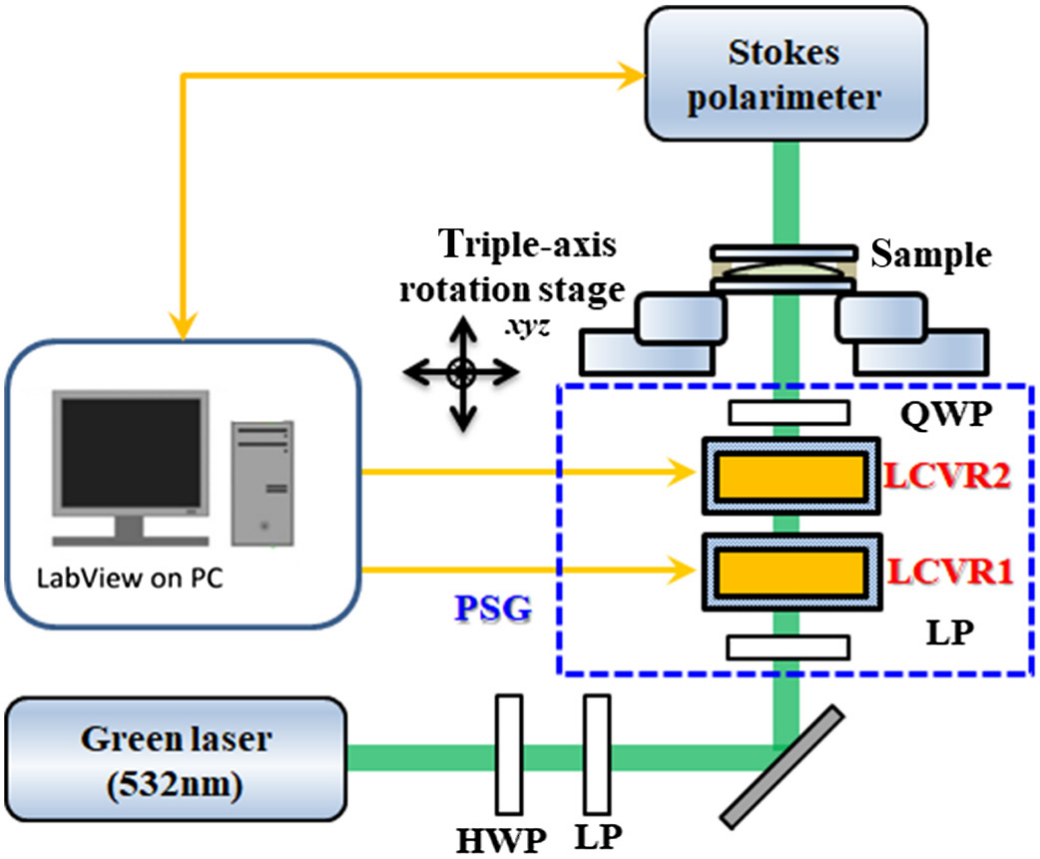
Schematic of the dual-retarder Mueller polarimetry system. The blue dashed lines represent the PSG unit employing an LP, two LCVRs (LCVR1 and LCVR2), and a QWP.

Figure 2 shows the dynamic measurement system proposed in this study. The system consists of a 532-nm green laser light source, a half-wave plate with a linear polarizer (LP) for tuning the laser power, an LP with its principal axis adjusted to 45 deg with respect to reference axis, two liquid crystal variable retarders (LCVRs) (Thorlabs) with their fast axis adjusted to 90 deg and −45  deg, respectively, and a quarter-wave plate (QWP) with fast axis adjusted to 90 deg. Here, we note that an LP in the PSG part is placed at the first optical component and defines an input polarization state that would have minimum interference between the tuning laser power part and the PSG part. According to this, we suggest that the principal axis of both LPs are set to the same direction for modulating the maximum power tuning range. When the misalignment occurs, it leads to becoming smaller power tuning range only. Finally, a commercial Stokes polarimeter (PAX1000, Thorlabs) having an accuracy of ±0.25% was used to measure the Stokes parameters of the state of polarization (SOP) of the sample at the focusing plane with about 3-mm light spot. For the PSG calibration, we used the Stokes polarimeter as a consistency reference coordinate to probe the alignment performance during each device setting, which way reduce misalignment effects. All types of polarization states could be generated by the different applied voltages, and the lookup table was created for measurements. According to our configuration and the calibrated lookup table of both liquid crystals, the same polarization state of the recorded signal by our commercial Stokes polarimeter is obtained under the condition without sample (air). Therefore, the generated polarization states were identified in this way before measuring the sample due to the reliable experimental data. As a result, four polarization states by modulating voltage could be produced for the calculations of the experimental Mueller matrix. A program designed in LabVIEW software (National Instruments) by our team was used to synchronously control the instrument through interfaces that were constructed in house. The program was also used to electronically control the two aforementioned liquid crystal devices, which could be used to rapidly produce any polarization state and record the SOP of the transmission light after passing through the sample.

### 2.3 Stokes–Mueller Matrix Polarimetry Formalism

The polarization state of the light passing through the sample was described in terms of four parameters of the Stokes vector (S), denoted as I,Q,U, and V ($S_{0}$, $S_{1}$, $S_{2}$, and $S_{3}$ with normalized total intensity, $I_{t}$) in the Cartesian coordinate system. The Mueller matrix (M) of a particular sample is a 4×4 matrix that provides a complete description of the polarizing and depolarizing properties of the medium for any polarization of the input light. In addition, the Mueller–Stokes formalism is defined by the linear relationship

$$S_{\mathrm{out}}={\mathrm{MS}}_{\mathrm{in}},$$ (1)

where $S_{\mathrm{out}}$ and $S_{\mathrm{in}}$ are the Stokes vectors of the output and the incident light, respectively. To yield all of the equations required to solve the sample matrix M, four polarization states of the input light were used, and the Stokes vectors need to form a tetrahedron in polarization space (on the Poincaré sphere) for the calculation condition,^35^ including three linear polarization states (with the angle of the major axis at 0 deg, 45 deg, and 90 deg) and one right-hand circular polarization state. The corresponding input Stokes vectors are given as $S_{\mathrm{in}\;0\;\deg}=[1,1,0,0{]}^{T}$, $S_{\mathrm{in}\;45\;\deg}=[1,0,1,0{]}^{T}$, $S_{\mathrm{in}\;90\;\deg}=[1,-1,0,0{]}^{T}$, and $S_{\mathrm{in}\_R}=[1,0,0,1{]}^{T}$, respectively. Here, we note that the selection of four input polarization states in this study is not the optimal tetrahedral for the set of input states. The configuration of the collimated transmission along the z direction of the propagation of light is determined as the differential matrix m corresponding to a macroscopic Mueller matrix M of the sample. The relationship is expressed as

$$m=(\frac{\mathrm{dM}}{\mathrm{dz}})M^{-1}.$$ (2)

Because the light transmits through a finite length of sample, the matrix contains the information by accumulated effects. In general, the optical path weighted differential matrix ($\overset{¯}{m}$) obtained from Eq. (2), as given in Ref. 29, is measured for the unknown path length of the homogeneous medium (Δz), which is expressed as

$$\overset{¯}{m}=\Delta z*m.$$ (3)

Based on the matrix of accumulated information, the parameters have a clear physical interpretation, such as linear birefringence, linear dichroism, circular dichroism, circular birefringence, and so on. According to the anisotropic form of collagen material, the linear phase retardation properties ($\overset{¯}{\beta }$) from the linear birefringence with accumulated effects are obtained as

$$\overset{¯}{\beta }=\Delta z*\beta =\sqrt{\left[\frac{{\overset{¯}{m}}_{42}-{\overset{¯}{m}}_{24}}{2}]+[\frac{{\overset{¯}{m}}_{34}-{\overset{¯}{m}}_{43}}{2}\right]},$$ (4)

where ${\overset{¯}{m}}_{ij}$ are the elements of the optical path weighted differential Mueller matrix of the sample.

## 3 Results and Discussion

### 3.1 Collagen Solution and Gel Matrix with Different Optical Path Length

**Fig. 3:**
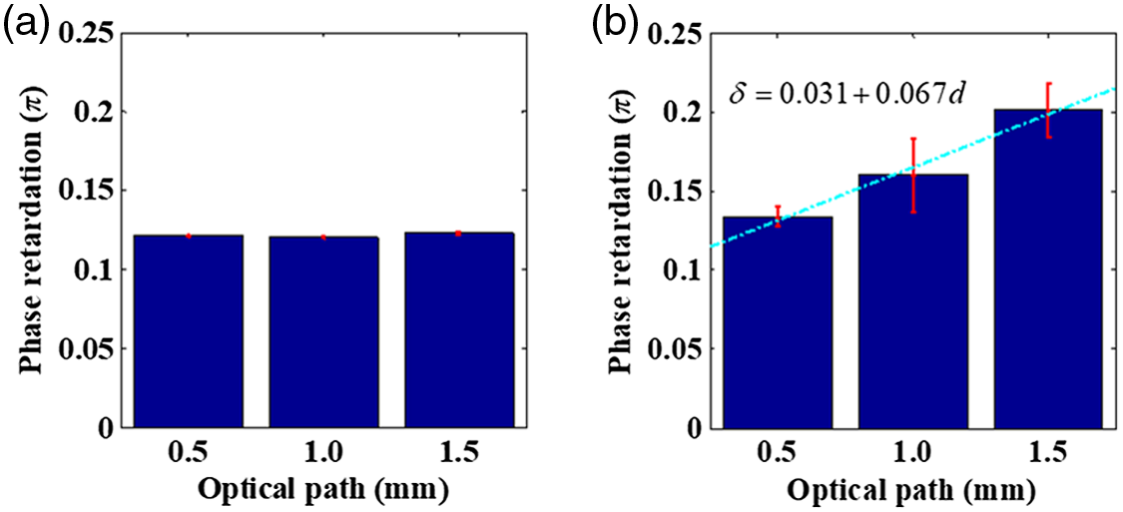
Linear retardance measurements for 2 mg/mL (a) collagen solution and (b) collagen gel. Varying thicknesses of the samples resulting in different optical path lengths are represented on the x axis. The error bars represent the standard deviation.

Based on the differential Mueller matrix method, linear phase retardation measurements for the constant-concentration collagen solutions and gels are shown in Figs. 3(a) and 3(b), respectively. For the different optical path length of collagen molecules solution, an average linear phase retardation value was statistically calculated, yielding a value between 0.1205 and 0.1231. For collagen gels, the variation in retardance values corresponding to the optical path length (distinct thickness), as shown in Fig. 3(b), significantly increases from thin to thick paths, leading to the retardance values increasing from 0.1339 to 0.2009 ($R^{2}=0.9848$ for fitting). The coefficients of variation for the solutions, 0.025% (0.5 mm), 0.055% (1.0 mm), and 0.086% (1.5 mm), as shown in Fig. 3(a), are all observed to be lesser than those of the gels, 0.618% (0.5 mm), 2.354% (1.0 mm), and 1.705% (1.5 mm), respectively, as shown in Fig. 3(b). It is suspected that the fluctuation in retardance values is higher for thicker samples owing to the random accumulation of the tissular scaled orientation distribution of collagen fibers/fibrils in the gel matrix.

### 3.2 Collagen Solution and Gel Matrix of the Different Concentration

**Fig. 4:**
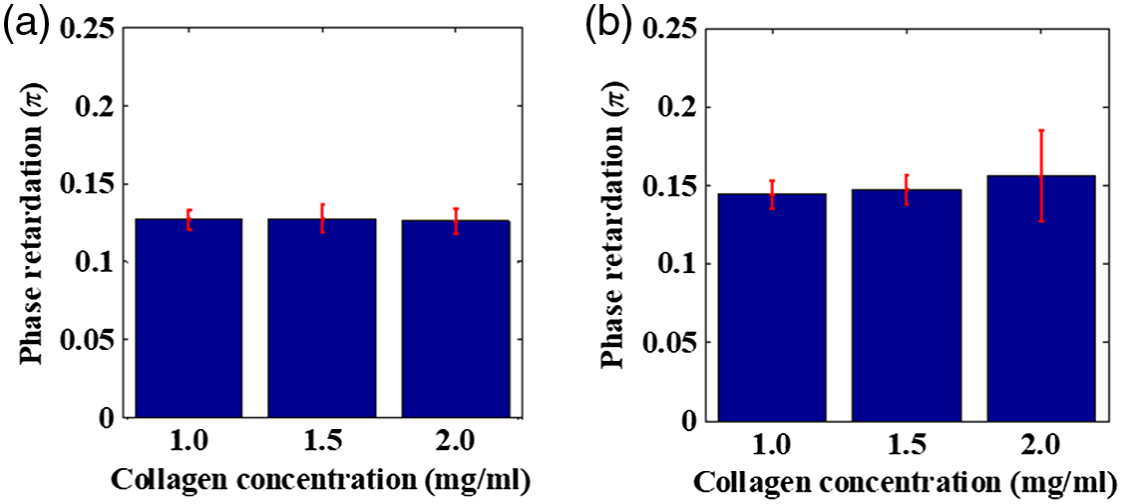
Linear retardance measurements for samples of equal thickness of (a) collagen solution and (b) collagen gel. Varying concentrations of collagen are represented on the x axis. The error bars represent the standard deviation.

In this study, we analyzed the linear phase retardation for three concentrations of collagen: 1, 1.5, and 2 mg/mL. An equal volume (200  μL) of collagen solution of each of the aforementioned concentrations was prepared in microwells. Along with collagen solutions, collagen gels were also prepared for measurement and analysis of the linear phase retardation, the results of which are presented in Fig. 4. As shown in Fig. 4(a), the retardance remained unchanged with increasing collagen concentration for collagen solutions and had an average value between 0.1260 and 0.1280. For collagen gels of different concentrations, averaged retardation values for 1, 1.5, and 2 mg/mL collagen gels were obtained as 0.1447±0.009, 0.1472±0.009, and 0.160±0.0236, respectively. The statistical results indicate that the increase in retardance was faster as the collagen concentration increased. The standard deviation of the measured linear retardance values of the 2 mg/mL collagen gel over four repeated tests was significantly high.

The results shown in Figs. 3 and 4 show the variation of retardance with differing thicknesses and concentrations of samples. According to this, we found an increasing retardance fluctuation with increasing self-assembly of collagen monomer concentrations into fibrils or thickness fluctuations (optical path length), which is similar to previous findings.^19^^–^^22^^,^^32^

### 3.3 Glycation Reacting with Different Concentration

**Fig. 5:**
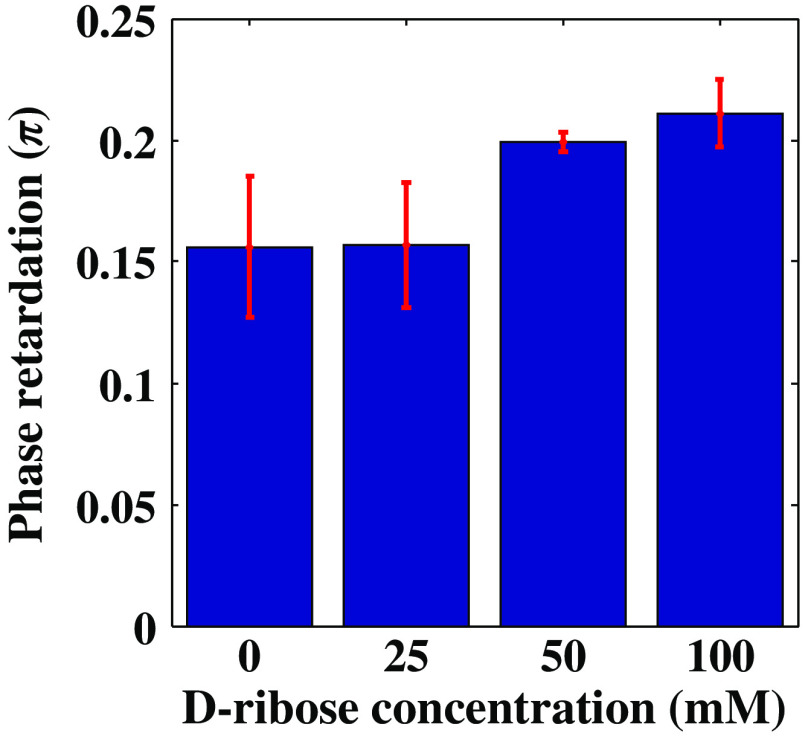
Linear retardance measurements for glycated collagen gel. Varying concentrations of D-ribose after 4 days are represented on the x axis. The error bars represent the standard deviation.

A further series of experiments was performed to investigate the protein glycation effect of collagen by measuring the variation in the phase retardation of collagen gel with different concentrations of D-ribose solutions after an incubation period of 4 days. Before nonenzymatic process, the collagen gels initially were prepared at the same time and the same condition with equal concentration of collagen (2 mg/mL) to exclude the impact of collagen gel thickness fluctuations. The gels were incubated and were maintained at ∼37°C during glycation treatment. Figure 5 shows the experimental results obtained for gels having D-ribose added at concentrations of 0, 25, 50, and 100 mM, yielding average phase retardations of 0.1560, 0.1568, 0.1995, and 0.2110, respectively. Thus, the collagen gel with the highest concentration of D-ribose was observed to show a 35% increase in linear retardance in comparison with that of the gel without D-ribose. In addition, the results show that collagen gel reacts with higher concentrations of D-ribose in a manner that leads to lesser variation in the retardance value. Overall, a statistically significant difference was induced by the higher D-ribose concentration in phase retardation of the glycated collagen gel compared with that of the PBS control group (0 mM concentration of D-ribose).

## 4 Discussion

In this study, Stokes–Mueller matrix polarimetry was implemented by electronically modulating the polarization state generator (PSG) to produce all types of polarization states without moving any part and by detecting the signal directly using a Stokes polarimeter. The PSG can produce any arbitrary SOP using two liquid crystal retarders and a QWP setup, which was proposed by Morales et al..^36^ The details of this setup were elaborated on in our previous paper.^37^ The dual-retarder setup is more compact, rapid, and easily accessible, which led us to use this method for our experimentation. We obtained results based on performing measurements for linear phase retardance to characterize solution and gel forms of collagen via differential Mueller matrix analysis. Our approach can thus be applied as a nondestructive biophotonic probing technique for studying native and glycated type I collagens. The values of the parameters in Eq. (4) are accumulated along the optical path (Δz), and the resultant intrinsic birefringence is expressed in radians. The index difference Δn=ny−nz is often referred to as the birefringence of the material, and the effective birefringence here is obtained from linear phase retardance (β) of optical samples in a form described in Ref. 38:

$$\Delta n=\beta \frac{\lambda }{2\pi \Delta z},$$ (5)

where λ is the wavelength of light.

Tissue birefringence has been probed using various methods in previous studies, which suggest that intrinsic birefringence occurs in regularly arranged fibrillary tissues, and the intrinsic birefringence for native collagen from tendons was measured to be in a range from $2.8\times 10^{-3}$ to $3.7\times 10^{-3}$.^39^^,^^40^ Our results show an initial estimate of the effective birefringence (Δn) for a collagen (Col I) gel with random fibers having ∼2% porosity to be $0.0178\times 10^{-3}$. This value is lower than expected because the collagen matrix is hydrated, which reduces the mass fraction and distorts the alignment, unlike native collagen tissue that has a high mass fraction and good alignment. The porosity of the collagen gel was obtained by calculating the ratio of the apparent collagen signal volume to the total volume using second-harmonic generation (SHG) microscopy, the details of which are elaborated on in our previous paper.

Our results in this paper are similar to those of other studies^8^^,^^15^ with regard to showing that the effective birefringence (phase retardation) of collagen in the glycated condition increases due to cross-linking among fibers or fibrils in the intermolecular space proportional to ribose concentration in the culture medium. This increase was not observed for controls and 25 mM gels. Under similar incubation conditions, Roy et al. used attenuated total reflectance-FTIR spectroscopy to demonstrate that the accumulation of fluorescent-AGEs showed direct linear proportionality to the ribose concentration.^10^

To our knowledge, this is the first study to quantify the birefringence of type-I collagen gels incubated with a reducing sugar. This indicates that our newly proposed approach is capable of assessing a similar concentration-dependent dose response in the amount of bound collagen in cross-linking reactions without having to detect AGE formation for nonenzymatic glycation processes. Further, this method creates great potential for studying reversible/irreversible cross-linking of collagen in tissue, as well as for developing methods to distinguish altered organization of collagen among different stages of the glycation process, especially in diabetes.

In conclusion, we showed that the amount of collagen fibrillar/fiber network in a gel matrix can be reliably determined from the measurement of the optical phase retardance ($\overset{¯}{\beta }$), yielding an effective linear birefringence obtained using a decoupled analytical technique based on the Stokes–Mueller matrix differential method. Results obtained by analysis of the collagen gel matrix in our experiments were consistent with those of previous studies and enabled the differentiation of the fiber cross-linking state for a nonenzymatic glycation process. In particular, this label-free and contact-free approach provides a quick and simple modality, which can have important applications for further studies on glycated collagen in biological samples. Hence, analysis facilitated by this method, which does not involve slicing and staining the tissue, represents a powerful tool that can provide new insights into early state clinical diagnostics in terms of time and cost efficiency, as well as help to track the development of diabetes and similar diseases.
